# Identification of lactylation-associated immune and metabolic regulators in bladder cancer via integrated bulk and single-cell transcriptomics

**DOI:** 10.3389/fimmu.2025.1604758

**Published:** 2025-07-09

**Authors:** Yanjun Chen, Yu Sun, Xiaoyan Liu

**Affiliations:** ^1^ Department of Cardiac Intensive Care Unit, the Cardiovascular Hospital, the Affiliated Guangdong Second Provincial General Hospital of Jinan University, Guangzhou, China; ^2^ Department of Hematology, Sun Yat-sen Memorial Hospital, Sun Yat-sen University, Guangzhou, China; ^3^ Guangdong Provincial Key Laboratory of Malignant Tumor Epigenetics and Gene Regulation, Sun Yat-Sen Memorial Hospital, Sun Yat-Sen University, Guangzhou, China

**Keywords:** lactylation, immunological regulators, bladder cancer, single cell, metabolic regulators

## Abstract

**Background:**

Lactate-driven metabolic reprogramming and histone lactylation play pivotal roles in bladder cancer (BLCA) progression, yet their underlying mechanisms and regulatory genes remain poorly understood.

**Methods:**

Using transcriptomic data from The Cancer Genome Atlas (TCGA), we identified lactylation-associated genes and constructed a prognostic signature. Comprehensive bioinformatics analyses were conducted to assess immune infiltration, tumor microenvironment characteristics, and the lactylation landscape at the single-cell level. Furthermore, we performed *in vitro* experiments to evaluate the biological functions of key lactylation-related genes in BLCA cells.

**Results:**

Six lactylation-related hub genes were identified, among which FASN and RUNX2 were significantly upregulated in BLCA and associated with poor prognosis. Single-cell analyses revealed elevated lactylation signatures in tumor epithelial and immune cells. Knockdown of FASN or RUNX2 in BLCA cell lines significantly suppressed cell proliferation, induced apoptosis, and reduced intracellular lactate levels. Correspondingly, global protein lactylation was diminished, with dominant modification signals observed around 40 kDa, indicating a potential set of non-histone proteins as key functional targets.

**Conclusions:**

Our study highlights a metabolic-enzymatic axis wherein FASN and RUNX2 regulate lactate-driven protein lactylation in BLCA. These findings provide new insights into the non-histone functions of lactylation and suggest potential therapeutic targets at the intersection of metabolism and tumor immunity.

## 1 Introduction

By 2020, bladder cancer (BLCA) was anticipated to cause 573,000 cases of diagnosis and 213,000 mortality worldwide (1). The integration of surgical procedures and adjuvant chemotherapy has enhanced rates of survival in bladder cancer patients. The frequency of recurrence and aggression of BLCA substantially impact its unfavorable prognosis (2). Consequently, comprehending the molecular regulation network of BLCA is essential for enhancing treatment approaches. Under normoxic conditions, malignancy cells predominantly employ glycolytic metabolism rather than oxidative phosphorylation for generating energy, a phenomenon referred to as aerobic glycolysis or the Warburg effect (3). The Warburg effect is intricately linked to the advancement and aggressiveness of BLCA (4). Elevated glucose utilization, ultimately transformed into lactate via an accelerated glycolytic pathway regardless of oxygen presence, fulfills the energy requirements of fast cancer cell multiplication (5). The shift from oxidative phosphorylation, which is the predominant metabolic pathway in most healthy cells, to glycolysis leads to a substantial increase in lactate production (6).

Lactate was traditionally viewed as a minor byproduct of glycolysis; however, in 2019, Zhao et al. revealed that lactate-induced lactylation of histone lysine residues constitutes an epigenetic alteration that directly enhances gene transcription in chromatin (7, 8). In 2022, Ye et al. improved the detection of protein lactylation with tandem mass spectrometry, demonstrating its extensive prevalence and considerable influence on non-histone protein activities (9). In 2023, Yuan et al. identified that the lactylation of meiotic recombination protein 11 (MRE11) is crucial in conferring resistance to chemotherapy (10). Recent *in vitro* studies suggest that elevated lactate levels hinder T-cell migration and promote the production of increased amounts of pro-inflammatory cytokines, such as IL-17, thereby sustaining local chronic inflammation (11). In BLCA, similar to numerous other malignancies, heightened lactate production is contingent upon the overexpression of critical elements in the glycolytic pathway such as glucose transporters (GLUTs) and lactate dehydrogenase isoform A (LDHA). These factors are considered promising targets for the development of novel therapeutic (12). Furthermore, to avert cell death caused by lactate accumulation, cancer cells enhance the expression of monocarboxylate transporters (MCTs) to expel this molecule. MCTs 1 and MCTs 4, in conjunction with their partner CD147, are frequently overexpressed in cancer cells and correlate with tumor aggressiveness (13). Consequently, increased lactate levels may be associated with the advancement of BLCA; however, its exact role necessitates more clarification. The expression and activity of lactate-associated genes in bladder cancer (BLCA) are hardly studied.

This study conducted a comprehensive analysis of RNA sequencing and single-cell RNA sequencing (scRNA-seq) data to identify key genes associated with lactylation in bladder cancer (BLCA) patients. Additionally, the relationship between the BLCA immune microenvironment (IME) and lactylation levels was elucidated. An innovative method shows potential for discovering biomarkers and directing further investigations into the mechanisms behind BLCA development.

## 2 Methods

### 2.1 Data acquisition

We retrieved RNA sequencing data for BLCA along with clinical information from the TCGA database (https://portal.gdc.cancer.gov/). The “limma” package was used to compare the overall differences in gene expression between BLCA patients and normal tissues, and the “ggplots2” and “heatmap” packages were used to show the results.

### 2.2 Lactylation-related hub gene identification in BLCA patients

Gene Ontology (http://geneontology.org//) identified 125 lactylation-related genes, of which 47 exhibited significant expression alterations between BLCA patients and normal tissues. Subsequently, these genes were incorporated into other studies. Candidate gene screening often utilizes the least absolute shrinkage and selection operator (LASSO), a form of multivariate linear regression. It optimizes the model to mitigate covariance and overfitting by persistently reducing the coefficients through the incorporation of a punishment function. LASSO regression was employed utilizing the “glmNETs” R package to narrow the selection to 19 genes (14, 15). The Random Forest classifier and Support Vector Machine (SVM) analysis were implemented using “randomForest” and “kernlab.” R programs were utilized, and the 20 most significant genes were retained. The results of LASSO regression, Random Forest, and SVM studies were subsequently integrated to pinpoint the hub genes associated with lactylation. GO and Kyoto Encyclopedia of Genes and Genomes (KEGG) pathway analyses were conducted using the R package “clusterProfile.”

### 2.3 The connection between immunological characteristics and lactylation hub genes

We utilized the ssGSEA method with the R package “GSVA” to meticulously assess immune cell infiltration in each study sample. We subsequently analyzed the relationship between hub genes and infiltrating immune cells using Spearman analysis.

### 2.4 Gene set enrichment analysis

We analyzed the co-expression of hub genes using Spearman correlation. Gene set enrichment analysis was performed on the Hallmark gene sets (http://software.broadinstitute.org/gsea/msigdb/) using the R package “clusterProfiler,” concentrating on the significant co-expressed genes of hub genes (P < 0.05).

### 2.5 scRNA-seq analysis

We obtained scRNA-seq data from the GEO database (GSE222315), which includes samples from 9 BLCA patients and 4 normal surrounding tissues, and analyzed the data using the R tool “Seurat”. We excluded cells with mitochondrial gene expression exceeding 15%, ribosomal gene expression surpassing 3%, erythrocyte gene expression larger than 0.1%, and those expressing fewer than 200 genes or more than 7,500 genes to assure data quality. We alleviated batch effects among samples using the R package ‘harmony’ and standardized the data via the ScaleData function, followed by doing Principal Component Analysis (PCA) on the scaled data. We utilized the RunUMAP program for UMAP analysis. We utilized the FindAllMarkers program to discover differential genes within each cluster. We ultimately utilized the “SingleR” tool for cell type annotation.

We utilized the “GSVA” algorithm to evaluate the lactylation enrichment scores for each cell in the scRNA-seq dataset and to analyze each cell based on the HALLMARK pathway, KEGG_MEDICUS subset of Canonical pathways, and PID subset of CP Canonical pathways.

### 2.6 Cell culture and siRNA transfection

Human bladder cancer cell lines T24 and EJ-138 were obtained from the Center for Excellence in Molecular Cell Science, Chinese Academy of Sciences (Shanghai, China). T24 cells were cultured in RPMI-1640 medium, and EJ-138 cells were maintained in Minimum Essential Medium (MEM) (both from Procell, Wuhan, China), supplemented with 10% fetal bovine serum (FBS) (Procell, Wuhan, China) and 1% penicillin-streptomycin solution (Biosharp, Hefei, China). Cells were maintained at 37°C in a humidified incubator with 5% CO_2_. Small interfering RNAs (siRNAs) targeting FASN and RUNX2, as well as a negative control siRNA (siNC), were synthesized by GenePharma Co., Ltd. (Shanghai, China). Transfections were performed using Lipofectamine 2000 (Invitrogen, Carlsbad, CA, USA) following the manufacturer’s protocol. Knockdown efficiency was confirmed by quantitative reverse transcription PCR (qRT-PCR) and Western blotting.

### 2.7 Quantitative real-time PCR

Total RNA was isolated using TRIzol reagent (15596026, Life Technologies, USA) in accordance with the manufacturer’s protocol. For reverse transcription, 1 μg of total RNA was converted to cDNA using a PrimeScript RT reagent kit (RR037A, Takara, Dalian, China). Quantitative real-time PCR (qRT-PCR) was conducted with the TB Green Premix Ex Taq II (RR820A, Takara) on a real-time PCR detection system. Gene expression levels were normalized to GAPDH as the internal reference, and relative quantification was calculated using the 2^−ΔΔCt method.

### 2.8 EdU cell proliferation assay

Cell proliferation was evaluated using an EdU Apollo567 *In Vitro* Imaging Kit (RiboBio, Guangzhou, China) following the manufacturer’s protocol. Briefly, T24 and EJ-138 cells were seeded in 24-well plates and transfected with siFASN, siRUNX2, or negative control siRNA. After 48 hours, cells were incubated with 50 μM EdU solution for 2 hours at 37°C. Following incubation, cells were fixed with 4% paraformaldehyde, permeabilized with 0.5% Triton X-100, and stained according to the kit instructions. Nuclei were counterstained with DAPI, and EdU-positive proliferating cells were visualized and quantified using fluorescence microscopy.

### 2.9 Colony formation assay

T24 and EJ-138 cells were seeded in 6-well plates at a density of 500 cells per well after transfection with siFASN, siRUNX2, or control siRNA. Cells were cultured under standard conditions for 10–14 days until visible colonies formed. Subsequently, colonies were fixed with 4% paraformaldehyde for 20 minutes and stained with 0.1% crystal violet for 15 minutes at room temperature. After washing and air-drying, colonies containing more than 50 cells were counted manually or using ImageJ software for quantitative analysis.

### 2.10 Flow cytometric analysis of apoptosis

Apoptosis was assessed using a FITC Annexin V Apoptosis Detection Kit (556547, BD Biosciences, USA) according to the manufacturer’s instructions. T24 and EJ-138 cells were seeded into 6-well plates at a density of 1.0 × 10^5^ cells/mL, and transfected with siFASN, siRUNX2, or a negative control siRNA (siNC) after 24 h. Following 72 h of transfection, cells were collected by trypsinization (EDTA-free), washed twice with phosphate-buffered saline (PBS), and then stained with 5 μL FITC-Annexin V and 5 μL propidium iodide (PI) in the dark for 30 minutes. After incubation, 400 μL of binding buffer was added, and the apoptotic cells were analyzed using flow cytometry.

### 2.11 Intracellular lactate measurement

Intracellular lactate levels were quantified using the L-Lactic Acid (LA) Colorimetric Assay Kit (E-BC-K044-S, Elabscience, Wuhan, China) according to the manufacturer’s protocol. Briefly, T24 and EJ-138 cells were collected 48 hours post-transfection with siFASN, siRUNX2, or negative control siRNA. Cells were lysed in extraction buffer, centrifuged to remove debris, and the supernatant was incubated with assay reagents. Absorbance at 530 nm was measured using a microplate reader (BioTek, USA). Lactate concentrations were normalized to the negative control (siNC) group based on a standard curve.

### 2.12 Western blot analysis of protein lactylation

Total protein was extracted from T24 and EJ-138 cells using RIPA lysis buffer containing protease inhibitors. Protein concentrations were quantified by BCA assay (Thermo Fisher, USA). Equal amounts (20–30 μg) of protein were separated by SDS-PAGE and transferred onto PVDF membranes (Millipore). After blocking with 5% non-fat milk, membranes were incubated overnight at 4°C with a pan-anti-lactyllysine antibody (PTM-1401, PTM Bio, Hangzhou, China; 1:1000). After washing, membranes were incubated with HRP-conjugated secondary antibodies and visualized using enhanced chemiluminescence (ECL; Bio-Rad). GAPDH (1:5000, Cell Signaling Technology) was used as the loading control.

### 2.13 Statistical analysis

All bioinformatics analyses were conducted using R software (version 3.6.1). The association between the risk score and overall survival (OS) in BLCA patients was evaluated using univariate and multivariate Cox proportional hazards regression models. For *in vitro* experiments, data are expressed as mean ± standard deviation (SD), and statistical analysis was performed using GraphPad Prism 99.0 (GraphPad Software, San Diego, CA, USA). Each experiment was independently repeated at least three times, and p-values less than 0.05 were considered statistically significant.

## 3 Results

### 3.1 Overall expression profile in BLCA patients

**Figure 1:**
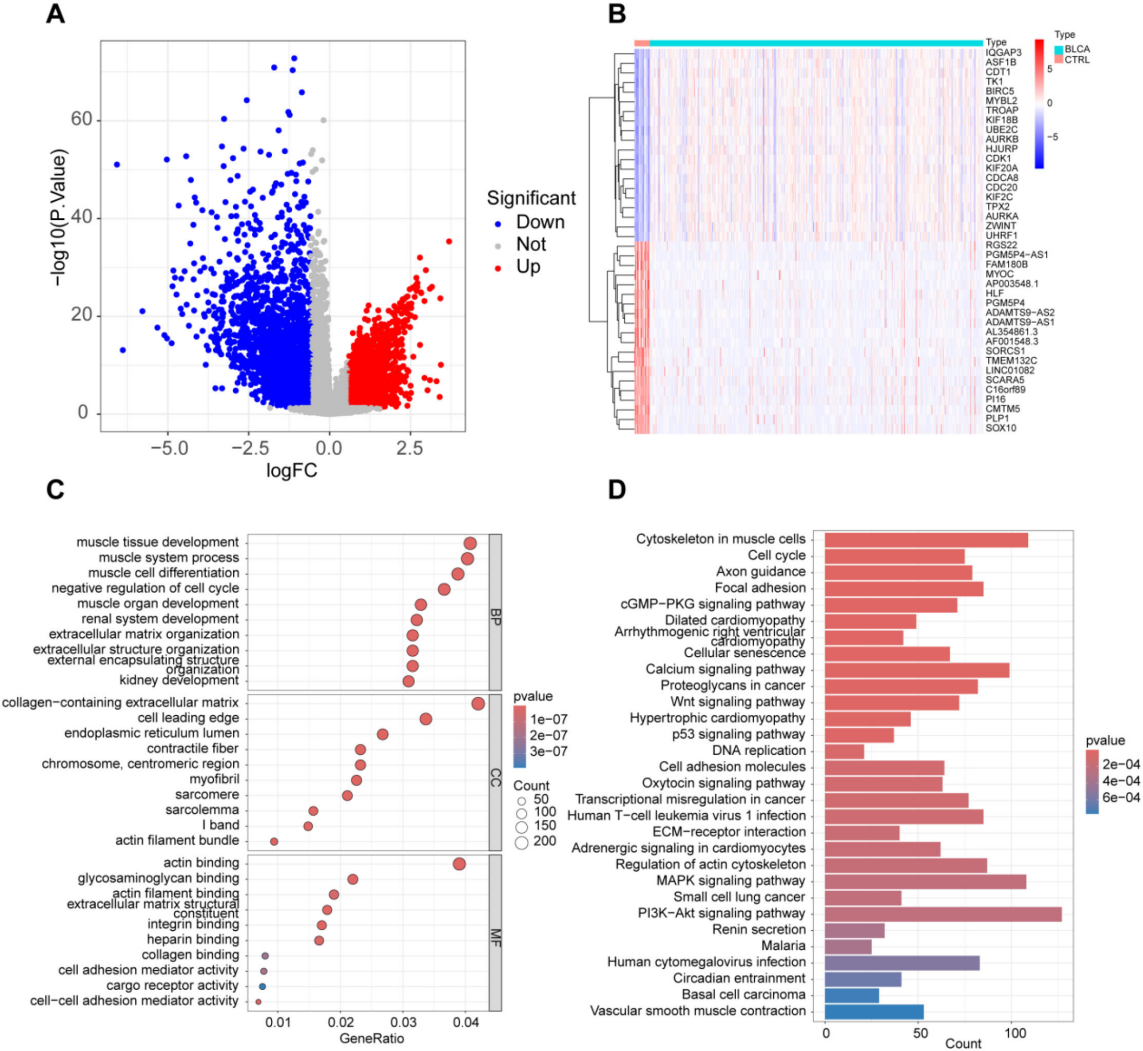
Overall expression profile in BLCA patients. **(A)** Analysis of variance volcano plots; **(B)** Heatmap displaying the top 20 genes with differential expression between BLCA and normal tissues; **(C)** GO functional clustering analysis; **(D)** KEGG functional clustering analysis.

Using gene expression datasets obtained from the TCGA database, we analyzed bladder cancer (BLCA) patients and healthy controls. Differential expression analysis revealed 984 upregulated and 1653 downregulated genes in BLCA patients (**Figures 1A, B**). Functional annotation and Gene Ontology (GO) analysis highlighted the significant involvement of “actin binding,” “glycosaminoglycan binding,” and “extracellular matrix structural constituent” in the pathogenesis of BLCA (**Figure 1C**). Additionally, KEGG pathway analysis identified key pathways associated with BLCA development, including “Cell adhesion molecules,” “PI3K-Akt signaling pathway,” and “MAPK signaling pathway” (**Figure 1D**).

### 3.2 Expression characteristics of lactylation-related genes

**Figure 2:**
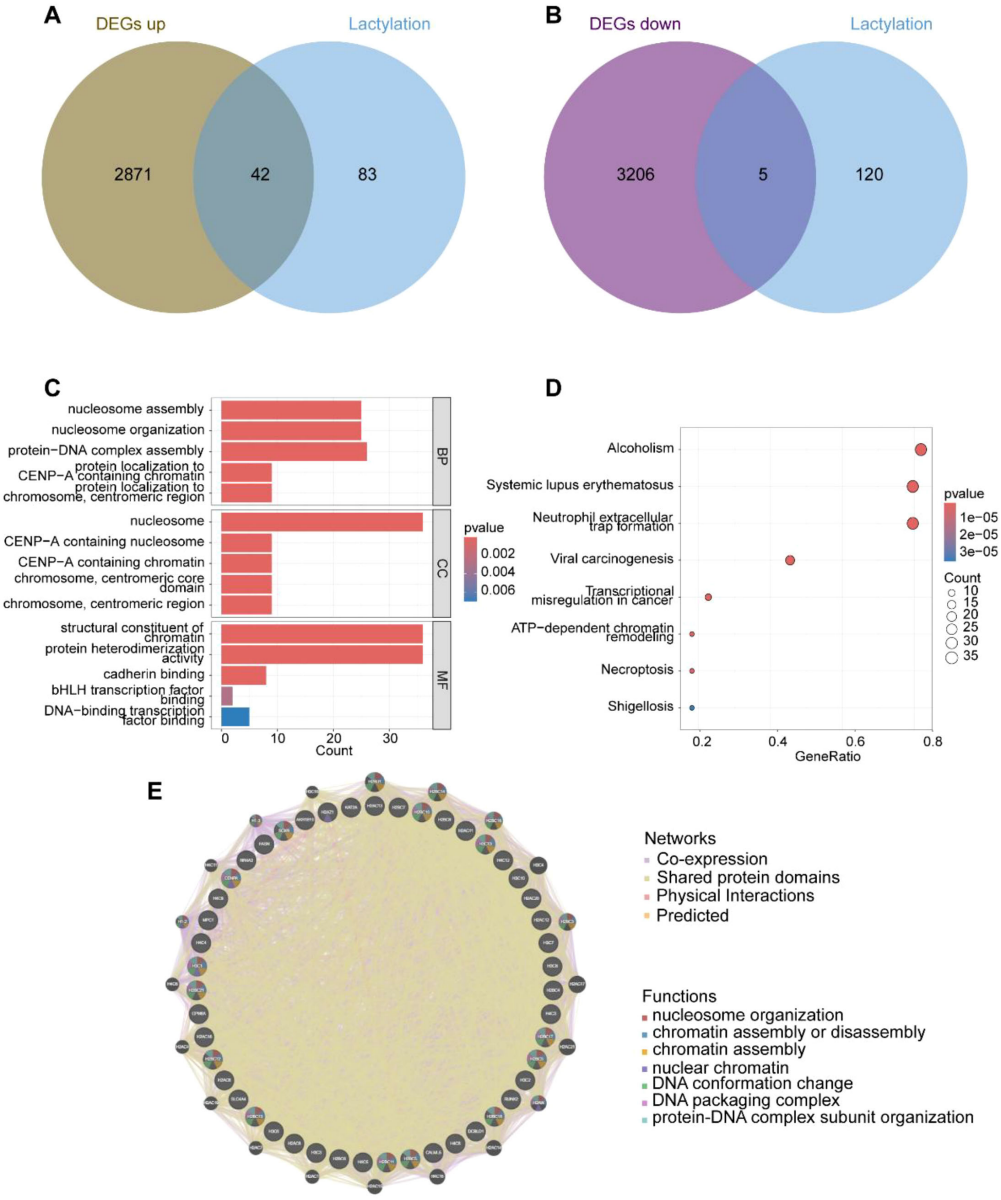
Expression characteristics of lactylation-related genes. **(A)** Up-regulated genes associated with lactylation; **(B)** Down-regulated genes associated with lactylation; **(C)** GO functional clustering analysis; **(D)** KEGG functional clustering analysis; **(E)** Gene interactions and functional analysis of the 47 up- and down-regulated lactylation-related genes.

After obtaining gene expression profiles, we analyzed the expression and differential patterns of 125 lactylation-related genes. Among these, 42 genes were significantly upregulated, while 5 genes were significantly downregulated in bladder cancer (BLCA) patients (**Figures 2A, B**, **Supplementary Figures 1A, B**). Functional analysis of these 47 differentially expressed genes (DEGs) revealed their involvement in critical biological processes. Gene Ontology (GO) analysis indicated significant alterations in “nucleosome assembly,” “protein-DNA complex assembly,” and “protein heterodimerization activity,” underscoring the pivotal role of epigenetic regulation in BLCA (**Figure 2C**). Furthermore, KEGG pathway analysis identified significant changes in pathways such as “Neutrophil extracellular trap formation,” “ATP-dependent chromatin remodeling,” and “Necroptosis,” all of which are closely associated with epigenetic regulation and immune modulation in cancer (**Figure 2D**). Finally, we mapped the interaction network of these 42 differentially expressed lactylation-related genes, providing a foundation for further investigation into their molecular mechanisms in BLCA (**Figure 2E**).

### 3.3 Identification of hub genes associated with lactylation

**Figure 3:**
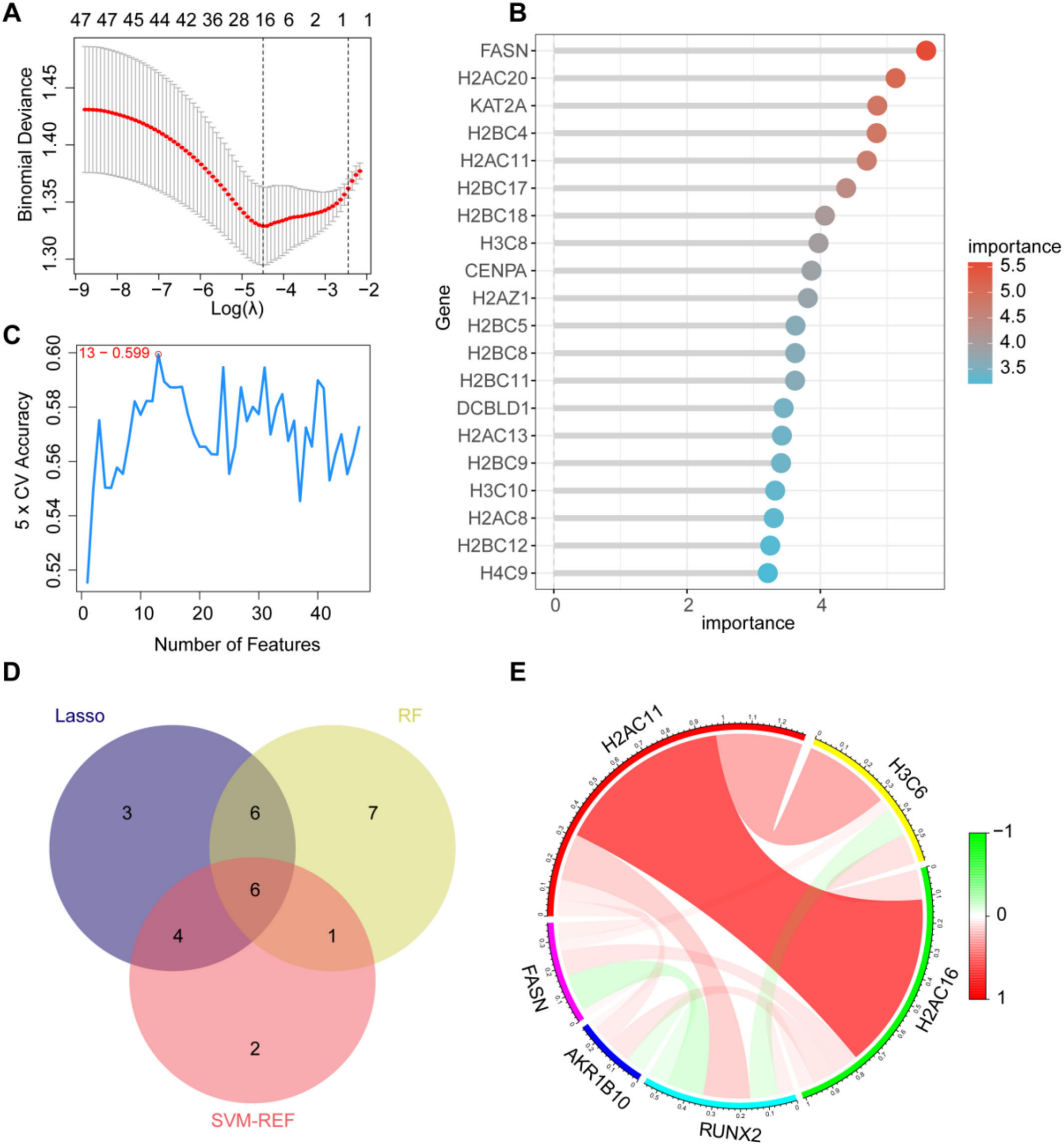
Identification of hub genes associated with lactylation. **(A)** LASSO regression to select 19 genes; **(B)** Random forest to identify 20 genes based on their importance; **(C)** SVM support vector machine to screen 13 genes; **(D)** Intersection of results from the three methods to identify six hub genes; **(E)** Correlation analysis of the six hub genes, with red indicating positive correlation and green indicating negative correlation.

To objectively identify the hub genes among lactylation-related genes, we performed LASSO regression analysis on 42 differentially expressed genes, narrowing down the candidates to 19 genes (**Figure 3A**, **Supplementary Figure 2A**). Subsequently, we utilized Random Forest Analysis and Support Vector Machine-Recursive Feature Elimination (SVM-RFE) to rank the lactylation-related genes, identifying the top 20 genes (**Figures 3B, C**, **Supplementary Figures 2B, C**). By integrating the results from these three methods, we ultimately identified six core lactylation-related hub genes: H2AC11, H3C6, H2AC16, RUNX2, AKR1B10, and FASN (**Figures 3D, E**).

The identification of these hub genes not only provides potential biomarkers for the diagnosis of bladder cancer but also establishes a foundation for further exploration of the role of lactylation in tumorigenesis.

### 3.4 Functional analysis of hub genes in BLCA patients

**Figure 4:**
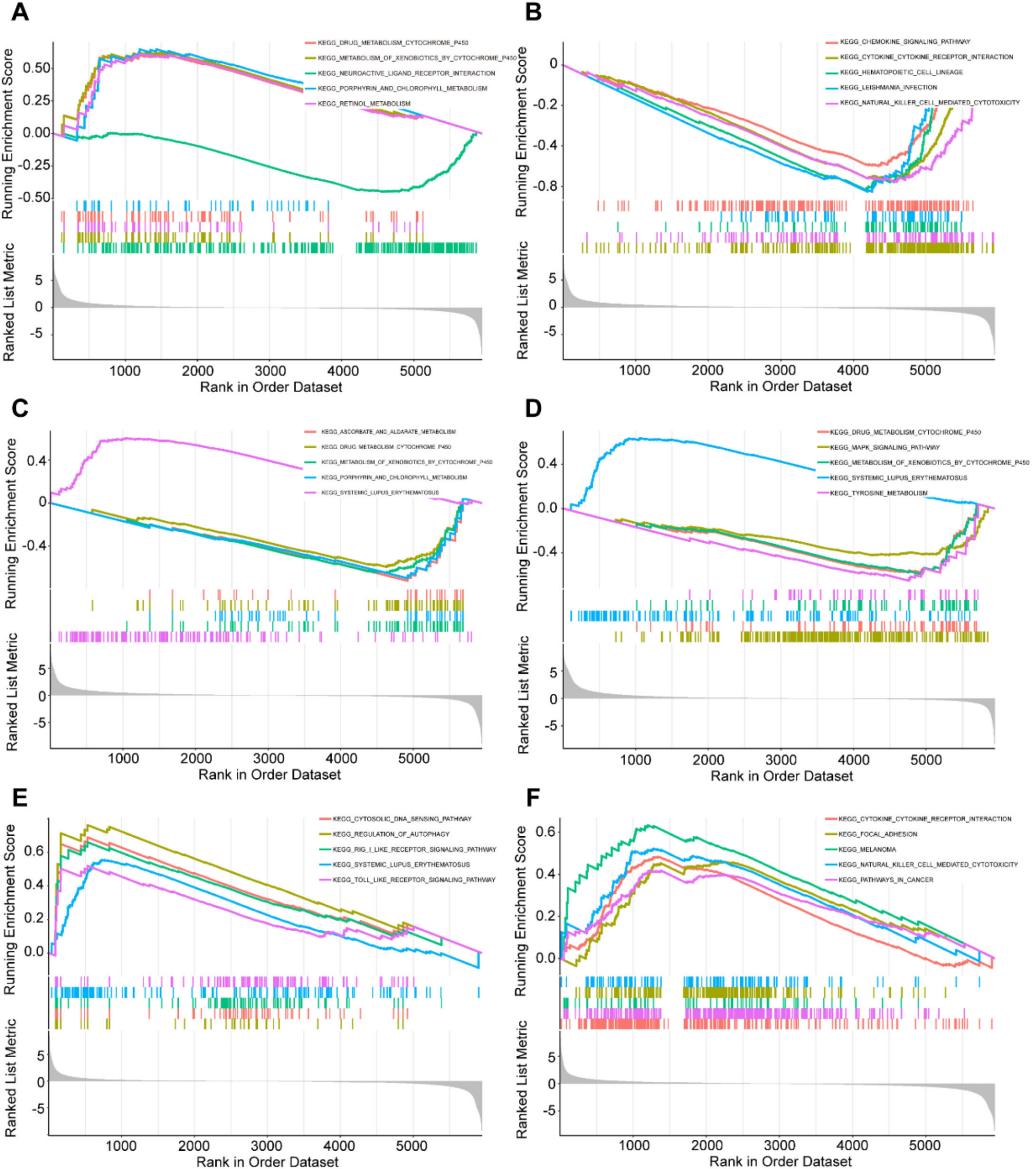
Functional analysis of hub genes in BLCA patients. The correlation between hub gene expression and KEGG pathway. The GSEA analysis of KEGG (top5) based on the correlation analysis result of **(A)** AKR1B10, **(B)** FASN, **(C)** H2AC11, **(D)** H2AC16, **(E)** H3C6 and **(F)** RUNX2.

Through genome-wide correlation analysis and Gene Set Enrichment Analysis (GSEA) of the six hub genes in bladder cancer (BLCA) patients, we identified their crucial roles in metabolism, immune regulation, and cancer-related pathways. Specifically, AKR1B10 expression was positively correlated with cytochrome P450 enzyme metabolism, while negatively correlated with the neuroactive ligand-receptor interaction pathway(**Figure 4A**). FASN expression showed positive correlations with chemokine signaling, cytokine-cytokine receptor interaction, and hematopoietic cell lineage pathways, but negative correlations with parasite infection-related pathways and natural killer (NK) cell-mediated cytotoxicity (**Figure 4B**). H2AC11 expression was positively associated with redox metabolism, such as ascorbate and porphyrin metabolism, while negatively regulating detoxification enzyme and immune-related pathways (**Figure 4C**). H2AC16 expression demonstrated significant positive correlations with the MAPK signaling pathway and tyrosine metabolism, but negative correlations with drug metabolism and immune disorder-related pathways (**Figure 4D**). H3C6 expression exhibited positive correlations with cytosolic DNA sensing, RIG-I-like receptor signaling, and Toll-like receptor signaling pathways, while showing negative correlations with systemic lupus erythematosus and autophagy regulation pathways (**Figure 4E**). Lastly, RUNX2 expression was positively correlated with cytokine-cytokine receptor interaction, focal adhesion, and cancer-related pathways, but negatively correlated with melanoma and NK cell-mediated cytotoxicity pathways (**Figure 4F**). These findings highlight the diverse functional roles of these hub genes in the pathogenesis of BLCA, providing a solid foundation for further exploration of their molecular mechanisms.

### 3.5 Expression of Hub genes is associated with immunological features in BLCA patients

**Figure 5:**
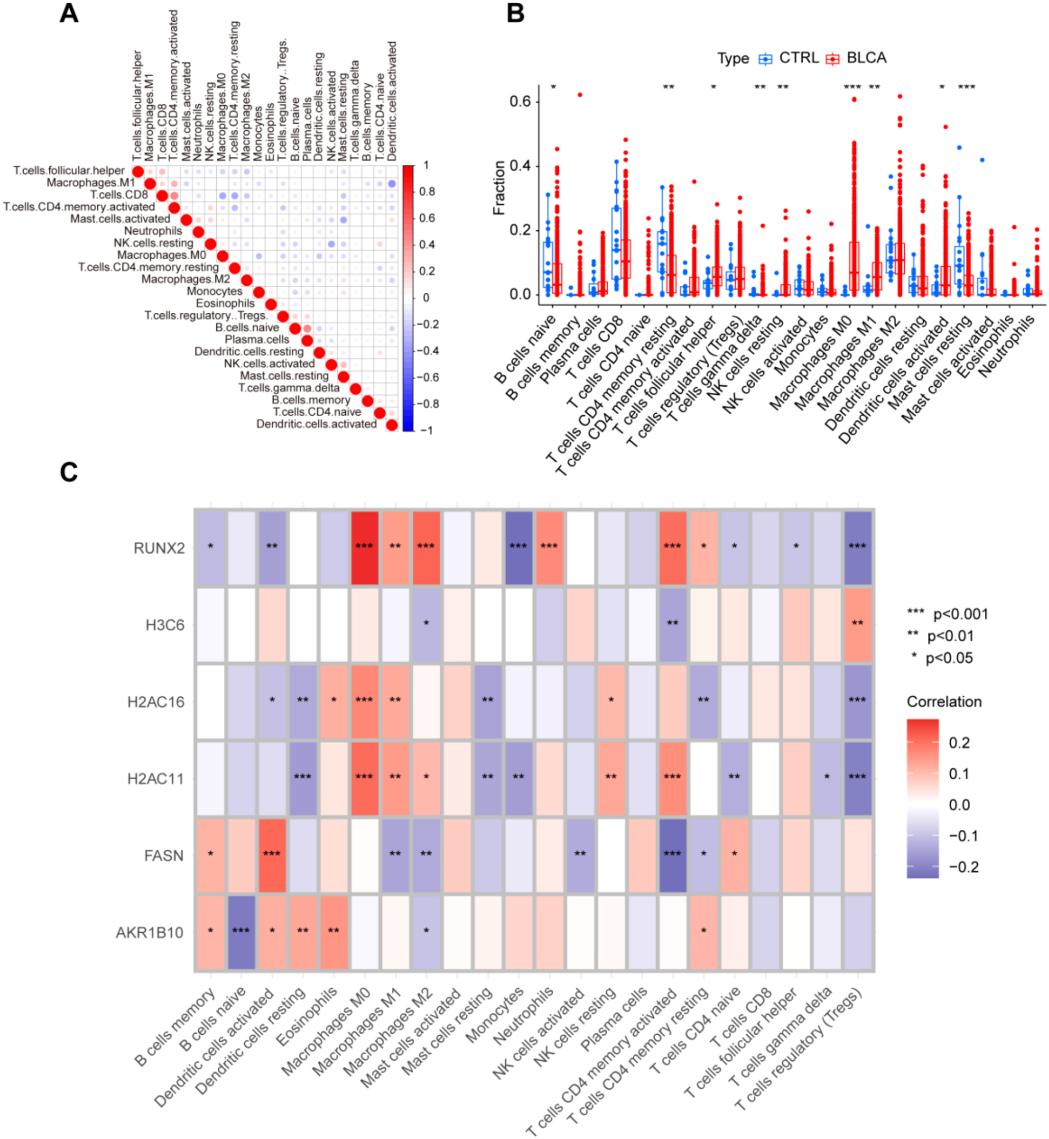
Expression of hub genes is associated with immunological features in BLCA patients. **(A)** Correlation of immune cell infiltration; **(B)** Differences in immune cell infiltration between BLCA and normal controls; **(C)** Correlation between immune cell infiltration and the six hub genes. *P < 0.05, **P < 0.01, ***P < 0.001, ns, not significant.

We examined the amounts of immune cell infiltration in tumor tissues compared to normal tissues. The results demonstrated a significant increase in the infiltration levels of 9 out of 23 immune cell types in BLCA patients (**Figures 5A, B**). RUNX2 expression displayed a significant negative correlation with the infiltration levels of six immune cell types, while showing favorable associations with macrophages, neutrophils, and memory CD4+ T cells. The expression of H3C6 showed a positive link with the infiltration of regulatory T cells, while indicating a negative correlation with M2 macrophages and activated memory CD4+ T cells. Moreover, the expressions of H2AC16 and H2AC11 showed a positive correlation with macrophages and resting NK cells, while revealing a negative link with resting dendritic cells, resting mast cells, and regulatory T cells. FASN expression showed a strong positive link with dendritic cell activation, whereas AKR1B10 expression revealed a notable negative correlation exclusively with naïve B cell infiltration (**Figure 5C**). The data indicate a robust link between hub gene expression and immune cell infiltration patterns in BLCA, underscoring their potential role in modulating the tumor immune microenvironment.

### 3.6 Identification of 24 cell clusters in BLCA patients using scRNA-seq data

**Figure 6:**
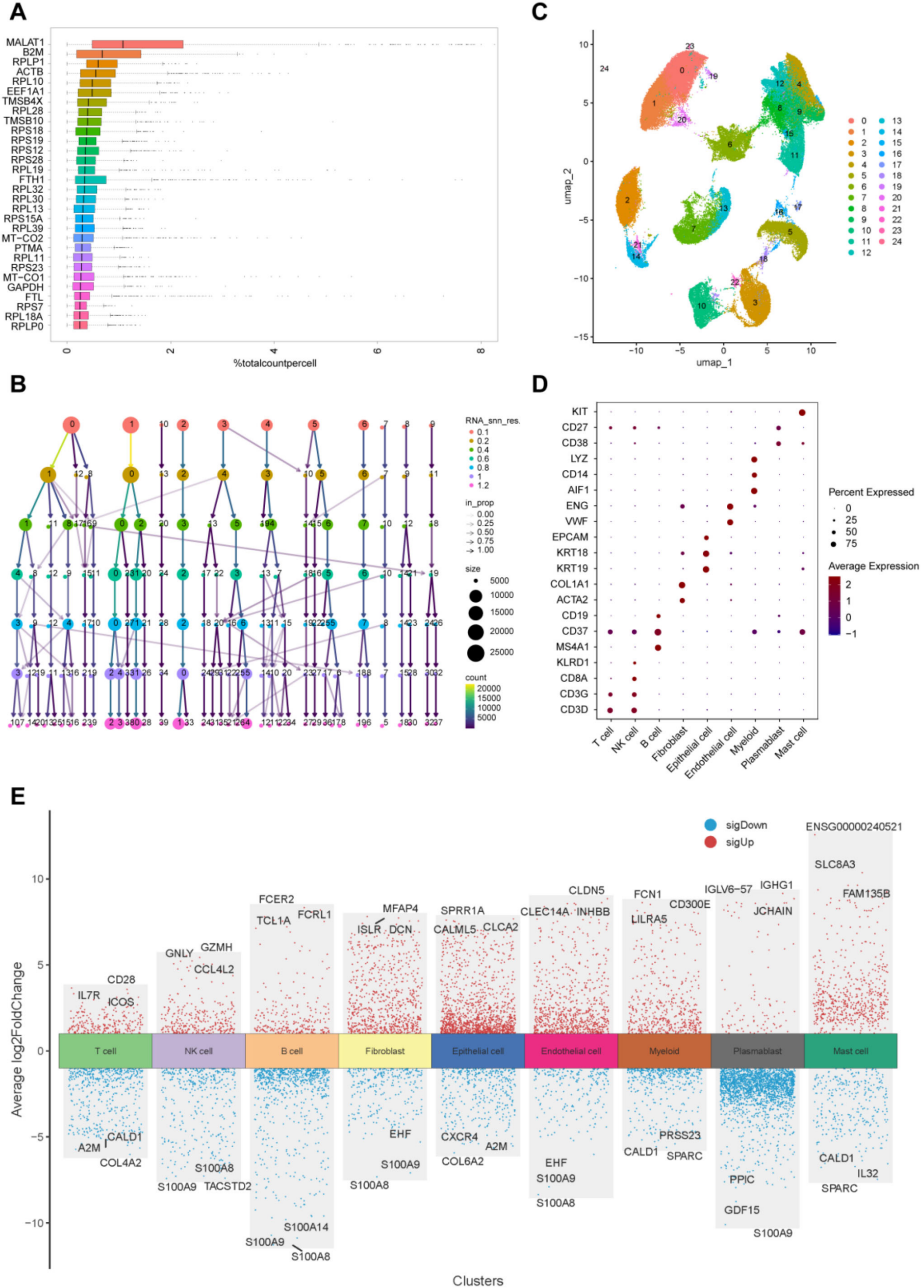
Identification of 24 cell clusters in BLCA patients using scRNA-seq data. **(A)** Expression of the top 30 genes; **(B)** Cluster dendrogram illustrating cell grouping at different resolutions; **(C)** UMAP plot showing 24 distinct cell clusters; **(D)** Dot plot displaying expression of top marker genes across the 9 cell types; **(E)** Top 3 differentially expressed genes per cluster.

Following quality control and normalization of scRNA-seq data from bladder cancer patients (GSE222315) (**Supplementary Figures 3A–C**), the top 25 genes are presented in **Figure 6A**. Dimensionality reduction was subsequently executed with Uniform Manifold Approximation and Projection (UMAP), thereby categorizing the cells into 24 discrete clusters (**Figures 6B, C**). The “SingleR” function was employed for cell annotation, resulting in the identification and categorization of eight cell types: T cells, B cells, NK cells, myeloid cells, epithelial cells, fibroblasts, endothelial cells, and plasmablasts (**Figure 6D**). Differential expression analysis was conducted to identify marker genes for each of the eight cell types (**Figure 6E**). These findings offer a detailed atlas of cellular heterogeneity in BLCA, establishing a basis for the subsequent investigation of cell-specific processes in bladder cancer.

### 3.7 scRNA-seq analysis validates the role of lactylation in immune interactions in BLCA

**Figure 7:**
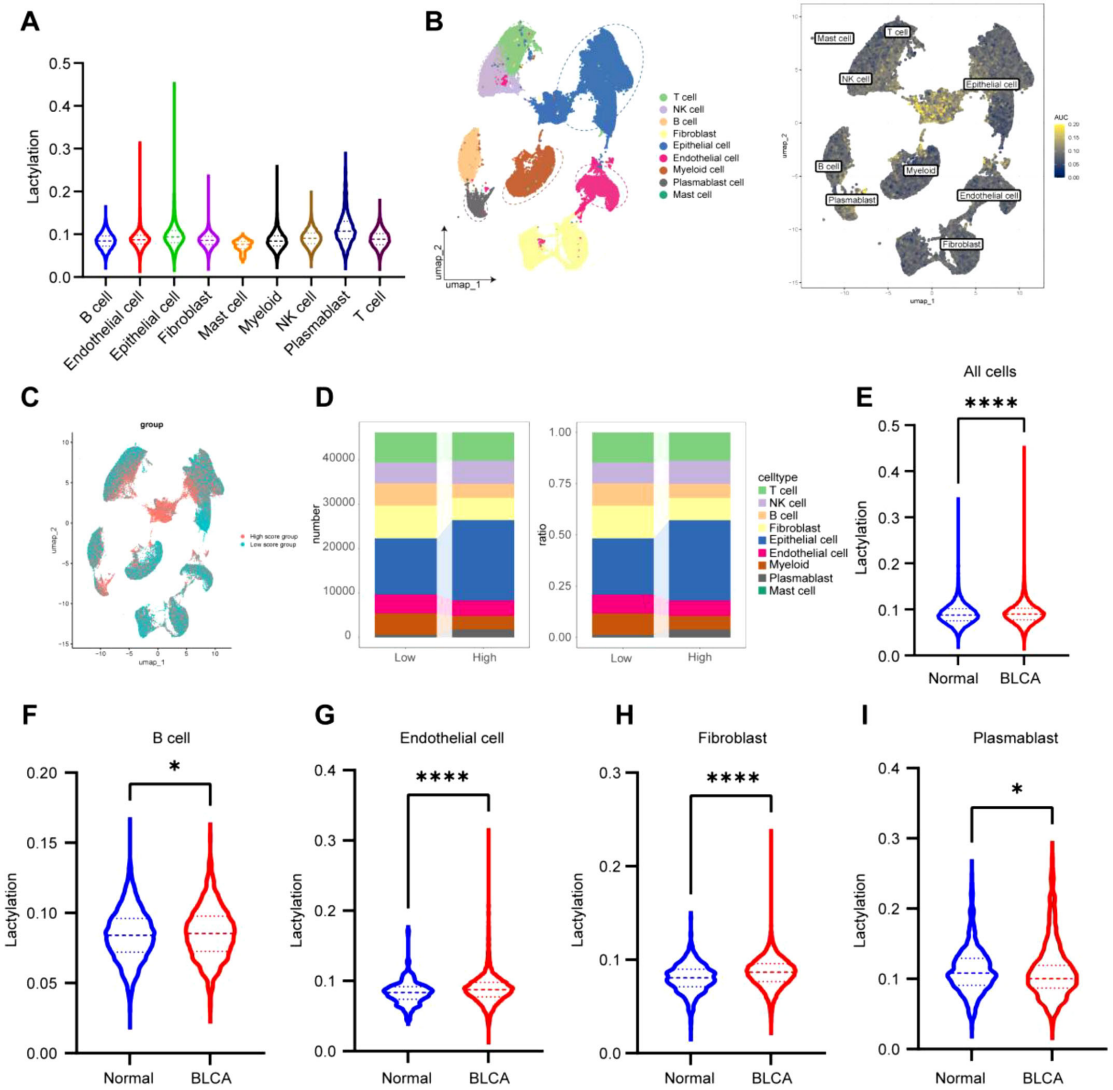
scRNA-seq analysis validates the role of lactylation in immune interactions in BLCA. **(A)** Scoring of individual cells based on the lactylation gene set using the R package GSVA, showing the distribution of scores across different cell types; **(B, C)** UMAP representation of lactylation gene set scores, categorized into high and low levels based on the median; **(D)** Number and percentage of cells from each type within the high and low lactylation gene set score groups; **(E)** Comparison of lactylation gene scores between BLCA tissues and normal tissues; **(F-I)** Variations in lactylation gene scores across different cell types. *P < 0.05, **P < 0.01, ***P < 0.001, ****P < 0.0001, ns, not significant.

We assessed the lactylation levels in BLCA by examining the expression profiles of 42 lactylation-related genes across several cell types at the single-cell level (**Figure 7A**). The findings indicated elevated lactylation scores in epithelial cells, T cells, and plasmablasts, whereas myeloid cells, fibroblasts, and endothelial cells displayed decreased scores (**Figure 7B**). Cells were subsequently categorized into high and low lactylation groups according to the median lactylation score (**Figure 7C**). Subsequent examination revealed that epithelial cells, NK cells, and plasmablasts exhibited elevated lactylation levels, whereas B cells, epithelial cells, fibroblasts, endothelial cells, and myeloid cells presented comparatively lower lactylation levels (**Figure 7D**).

Ultimately, cells were classified according to their origin from tumor or non-tumor tissues. The findings demonstrated that lactylation levels were markedly elevated in tumor tissues relative to non-tumor tissues (**Figure 7E**). Furthermore, several cell types, including B cells, endothelial cells, fibroblasts, and plasmablasts, demonstrated significantly increased lactylation levels in tumor tissues relative to their non-tumor equivalents (**Figures 7F–I**). These findings highlight the potential function of lactylation in facilitating immunological interactions within the tumor microenvironment of BLCA.

### 3.8 Functional clustering analysis identifies key pathways in infiltrating immune cells

**Figure 8:**
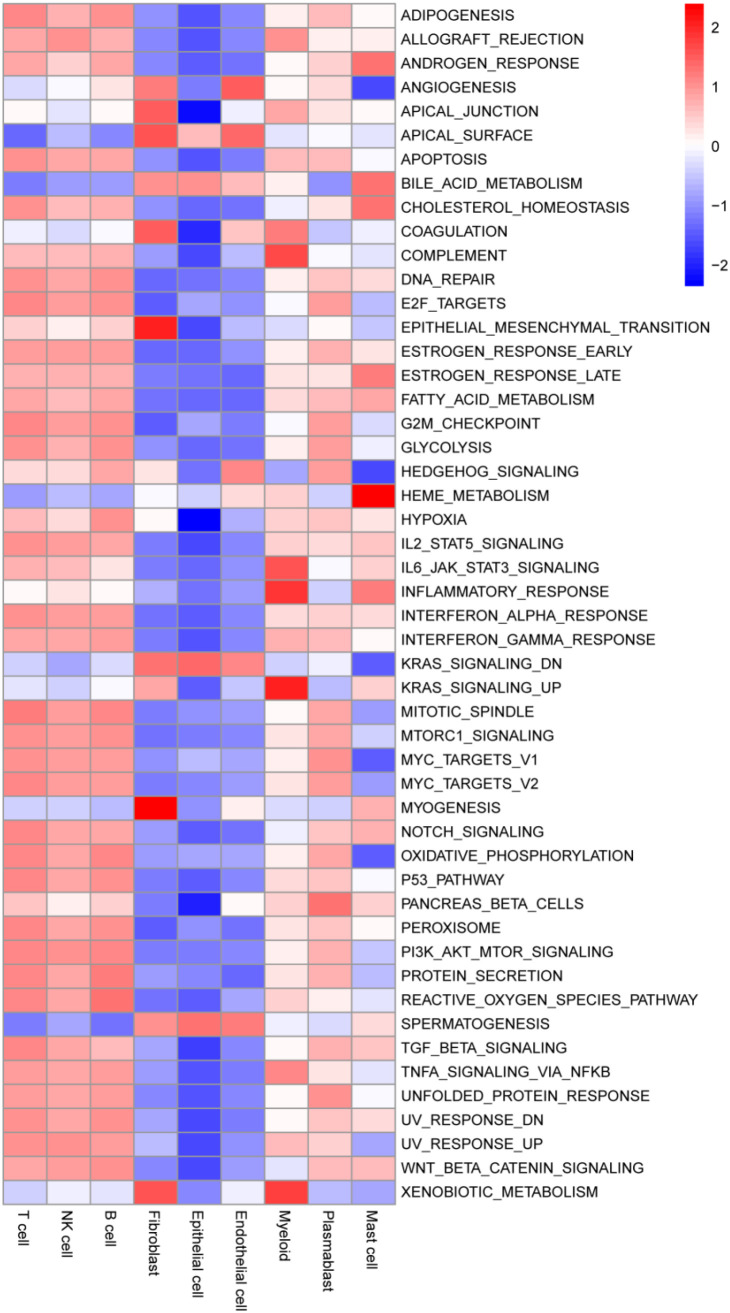
Functional clustering analysis identifies key pathways in infiltrating immune cells. Each cell was assigned a score based on the HALLMARK pathway from the MsigDB database.

To elucidate critical pathways linked to invading immune cells, we performed scoring of individual cells based on the MSigDB dataset using the R package GSVA and analyzed HALLMARK pathway scores across different cell types. The results demonstrated strong pathway enrichment consistency among fibroblasts, endothelial cells, and epithelial cells, where most pathways were negatively correlated, except for pathways such as “APICAL_SURFACE,” “BILE_ACID_METABOLISM,” “KRAS_SIGNALING_DN,” and “SPERMATOGENESIS.” In contrast, the remaining six immune cell types (particularly T cells, NK cells, and B cells) showed predominantly strong positive correlations across most pathways (**Figure 8**).

Similarly, pathway enrichment based on the KEGG dataset displayed comparable GSVA scoring patterns. Fibroblasts, endothelial cells, and epithelial cells exhibited negative correlations with most pathways, distinguishing them from the other six infiltrating immune cell types. Notably, mast cells achieved the highest enrichment scores in multiple pathways, such as “ALANINE_ASPARTATE_AND_GLUTAMATE_METABOLISM,” “ALPHA_LINOLENIC_ACID_METABOLISM,” and “ARACHIDONIC_ACID_METABOLISM” (**Supplementary Figure 4**).

Analysis of the PID dataset further supported the pathway enrichment patterns observed in GSVA scores. Fibroblasts, endothelial cells, and epithelial cells consistently showed similar pathway enrichments, where most pathways were negatively correlated, except for specific pathways such as “ALPHA_SYNUCLEIN_PATHWAY,” “BETA_CATENIN_DEG_PATHWAY,” “FGF_PATHWAY,” and “IL23_PATHWAY.” Conversely, the remaining six immune cell types, especially T cells, NK cells, and B cells, exhibited strong positive correlations across the majority of pathways (**Supplementary Figure 5**).

### 3.9 FASN and RUNX2 promote proliferation and inhibit apoptosis in BLCA cells

**Figure 9:**
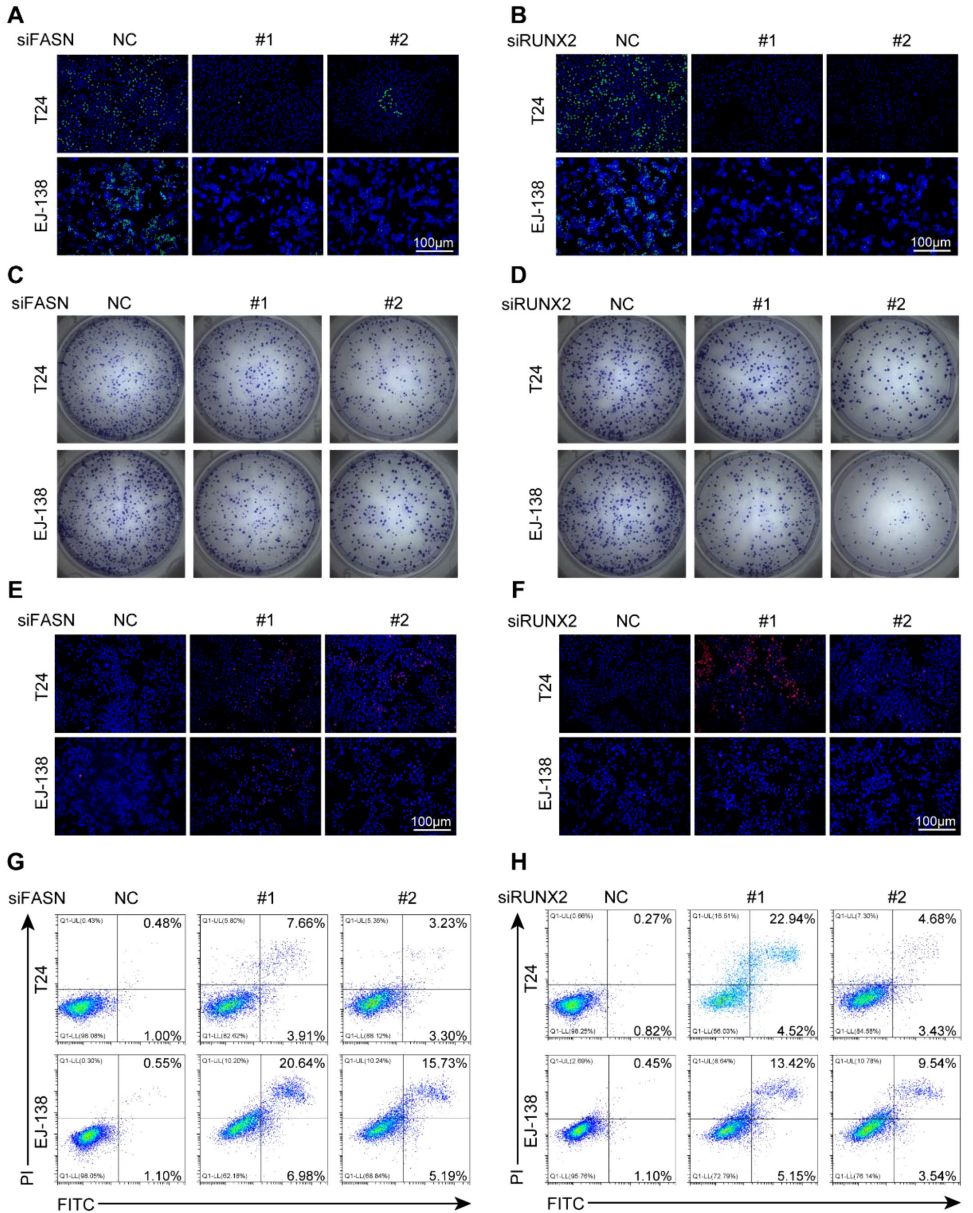
FASN and RUNX2 promote proliferation and inhibit apoptosis in BLCA cells. **(A-B)** EdU staining of T24 and EJ-138 cells transfected with siFASN **(A)** or siRUNX2 **(B)**. Green: EdU; blue: DAPI. **(C-D)** Colony formation assays after transfection with siFASN **(C)** or siRUNX2 **(D)**. **(E-F)** PI staining showing increased cell death after transfection with siFASN **(E)** or siRUNX2 **(F)**. **(G-H)** Flow cytometry analysis of apoptosis using Annexin V-FITC/PI staining following transfection with siFASN **(G)** or siRUNX2 **(H)**. All experiments were independently repeated at least three times. Scale bar: 100 μm.

We next investigated the differential expression of several target genes between normal bladder epithelial cells and BLCA cell lines. Compared to normal urothelial cells, AKR1B10 expression was markedly reduced, whereas FASN and RUNX2 were significantly upregulated in multiple BLCA cell lines (**Supplementary Figure 6A**). These findings suggested that FASN and RUNX2 might exert stronger oncogenic effects in BLCA cells. To further validate their functional roles, we explored the impact of FASN and RUNX2 on BLCA cell proliferation, survival, and apoptosis. We transiently transfected T24 and EJ-138 cells with siRNAs targeting FASN or RUNX2, as well as a non-targeting control siRNA. Knockdown efficiency was confirmed by qRT-PCR and Western blotting (**Supplementary Figures 6B–E**). We then assessed cell proliferation using EdU incorporation assays and colony formation assays. As shown in **Figures 9A–D** and **Supplementary Figures 7A–D**, silencing either FASN or RUNX2 significantly impaired the proliferative capacity of BLCA cells compared to controls. Subsequently, cell viability and apoptosis were evaluated using PI staining and flow cytometry. We observed a marked increase in apoptosis in BLCA cells following knockdown of FASN or RUNX2, indicating that both genes may promote tumor growth by conferring anti-apoptotic advantages (**Figures 9E–H**, **Supplementary Figures 7E-H**). Collectively, these results demonstrate that FASN and RUNX2 play critical roles in promoting the growth and survival of BLCA cells, underscoring their potential as therapeutic targets.

### 3.10 Silencing of FASN or RUNX2 suppresses intracellular lactate and protein lactylation in BLCA cells

**Figure 10:**
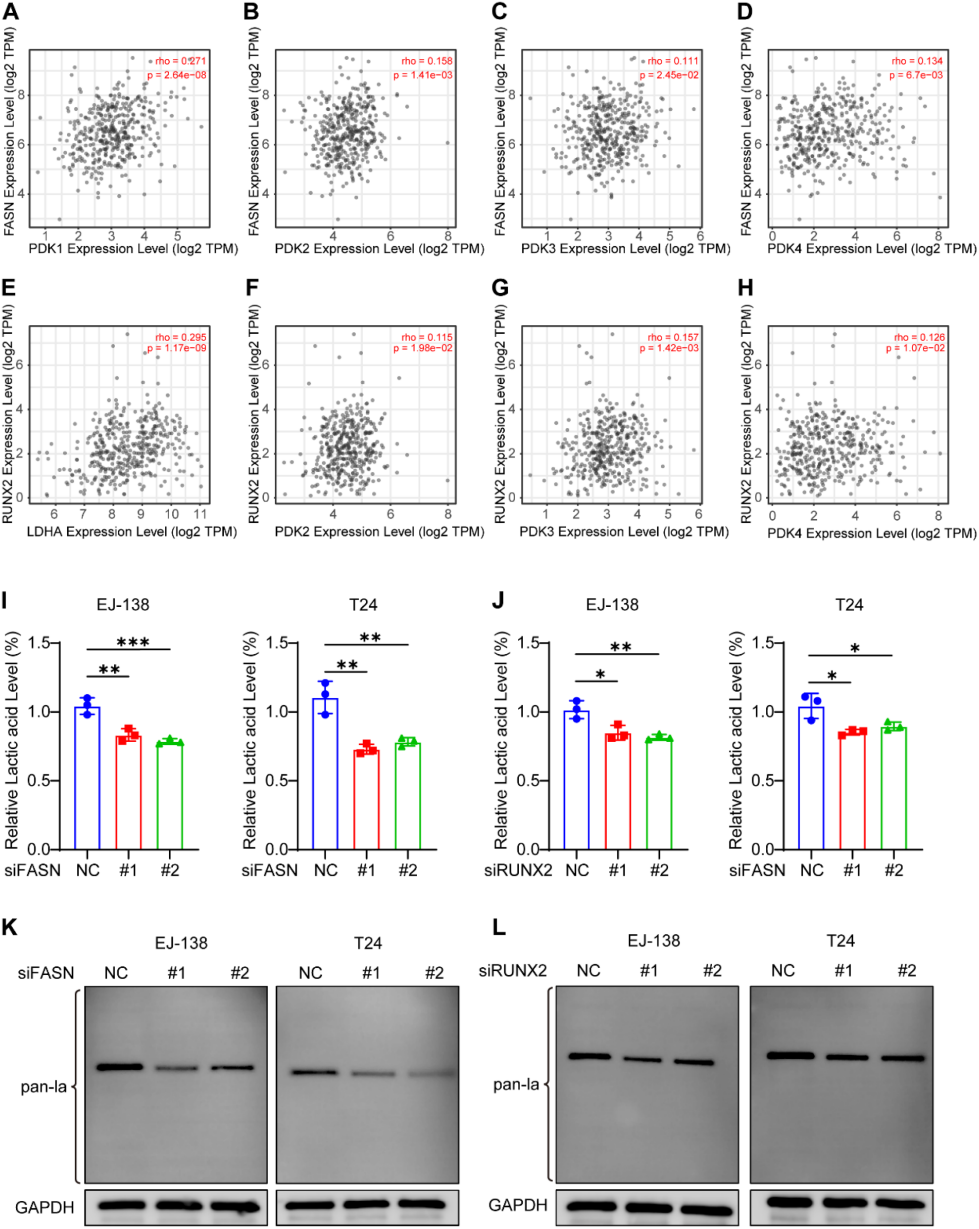
FASN and RUNX2 regulate intracellular lactate production and global protein lactylation in BLCA cells. **(A–H)** Spearman correlation analysis between FASN or RUNX2 and glycolysis-related genes (LDHA, PDK1, PDK2, PDK3) using TIMER2.0 database. **(I–J)** Intracellular lactate levels measured by L-lactic acid colorimetric assay in EJ-138 and T24 cells after siRNA-mediated knockdown of FASN **(I)** or RUNX2 **(J)**. Lactate concentrations were normalized to the negative control (siNC) group. **(K–L)** Western blot analysis of global protein lactylation in EJ-138 and T24 cells following knockdown of FASN **(K)** or RUNX2 **(L)**. Pan-lactylation signals were detected using anti-lactyllysine antibody, with GAPDH as the loading control. Dominant lactylation bands appeared near 40 kDa. *P < 0.05, **P < 0.01, ***P < 0.001, ns, not significant.

Although FASN and RUNX2 are annotated as lactylation-associated genes in Gene Ontology, their regulatory roles in lactate metabolism and protein lactylation remain unclear. To investigate potential upstream functions, we analyzed their correlations with key glycolytic enzymes using the TIMER2.0 database. Both genes exhibited moderate to strong co-expression with LDHA and members of the PDK family (**Figures 10A–H**), suggesting involvement in lactate biosynthesis.

To validate this, we measured intracellular lactate levels in EJ-138 and T24 BLCA cells following siRNA-mediated knockdown of FASN or RUNX2. Silencing either gene led to a notable reduction in lactate accumulation (**Figures 10I–J**), indicating a positive regulatory role in lactate production.

Given that lactate serves as a substrate for protein lactylation, we further assessed global lactylation by Western blot. Knockdown of FASN or RUNX2 significantly reduced pan-lactylation levels in both cell lines (**Figures 10K–L**). Notably, lactylation signals were predominantly observed around 40 kDa, suggesting that specific proteins within this range may be primary targets of lactylation in BLCA cells.

These results imply that FASN and RUNX2 modulate lactylation indirectly by promoting lactate generation. As both lactate accumulation and protein lactylation have been implicated in tumor growth and immune modulation, these genes may contribute to BLCA progression through metabolic-epigenetic pathways. However, further studies are needed to identify the modified proteins and clarify the downstream effects of lactylation in this context.

## 4 Discussion

The onset and advancement of cancer are marked by certain metabolic changes that facilitate the heightened proliferation of cancer cells. In BLCA, various metabolic pathways are altered to promote carcinogenesis. The transition to aerobic glycolysis, known as the Warburg effect, is typical of tumor cells, particularly in bladder cancer (BLCA). Elevated lactate levels and the acidity resulting from glycolytic metabolism contribute to cancer progression by enhancing invasiveness, acid-induced matrix degradation, and metastasis. Consequently, the Warburg effect is intimately linked to the advancement and intensity of BLCA. In this study, RNA sequencing data were utilized to identify six hub genes associated with lactylation, and the correlation between these hub genes and immune cell infiltration was analyzed. We conducted a comprehensive analysis of the scRNA-seq data and discovered that the level of lactylation in cells is strongly associated with the dysregulated activation of immune system functions. Analysis of RNA sequencing data revealed that the hub genes AKR1B10, FASN, H2AC11, H2AC16, H3C6 and RUNX2 may serve as potential markers to differentiate BLCA patients from healthy persons. AKR1B10, a member of the aldo-keto reductase superfamily, is essential for the conversion of carbonyl compounds, namely changing aldehydes or ketones into alcohols to aid in detoxification (16). Recent studies have indicated that AKR1B10 can promote histone lactylation, leading to the development of resistance in brain metastatic cells originating from lung cancer (17). Our study suggests that the expression of AKR1B10 in BLCA is positively correlated with the metabolism of various cytochrome P450 enzymes, indicating a potential critical role for AKR1B10 in epigenetic regulation. Further experimental research is required to elucidate its specific functions and underlying mechanisms. FASN (fatty acid synthase), a central enzyme in lipid metabolism, is frequently overexpressed in cancer cells, where it is linked to rapid proliferation and tumor progression (18, 19). Our study found that FASN is positively correlated with chemokine signaling and cytokine receptor interactions, which aligns with previous reports indicating that FASN mediates immune evasion and metastasis in tumor cells through the activation of fatty acid metabolism or by promoting palmitoylation (19, 20). Interestingly, our results also suggest that FASN may facilitate immune cell infiltration, implying that FASN contributes to the establishment of an immunosuppressive microenvironment rather than merely reducing immune cell infiltration.

H2AC11 and H2AC16, as members of the histone family, are primarily involved in chromatin remodeling and transcriptional regulation pathways (21, 22). However, we found that both genes also exhibit significant associations with various metabolic pathways. This suggests that their gene regulatory functions may primarily serve to remodel tumor metabolism. However, their roles in tumorigenesis and immune regulation have not been reported, warranting further investigation to explore their potential functions. Our study found that H3C6 is significantly correlated with multiple immune recognition and response-related signaling pathways, including the RIG-I-like receptor and Toll-like receptor signaling pathways. This suggests its potential regulatory role in anti-tumor immunity and immune evasion. Unfortunately, there is currently a lack of studies exploring the underlying mechanisms of its function. Runx2 is a DNA-binding transcription factor that modulates cell transformation by influencing multiple signaling pathways and the transcriptional activation of various downstream molecules in diverse physiological and pathophysiological contexts, including osteogenesis, malignancies, and vascular calcification (23, 24). However, the role of Runx2 in tumorigenesis and immune regulation has been rarely reported. Our study found that its expression is significantly correlated with cytokine-cytokine receptor interactions and cancer-related pathways, suggesting the potential value of Runx2 in tumor progression and immune regulation.

Metabolomics results indicate that immunological problems in BLCA patients are significantly linked to metabolic dysregulation. Utilizing scRNA-seq data, we conducted a thorough analysis of cellular lactylation levels in BLCA patients, revealing that lactylation levels at tumor tissues were markedly elevated compared to normal tissues, potentially linked to increased lactate accumulation at tumor locations. epithelial cells, T cells, and plasmablast cells had comparatively high overall lactylation levels; nevertheless, a considerable disparity in lactylation levels was seen between cells located at tumor and normal regions. Therefore, the regulation of lactylation in epithelial cells, T cells, and plasmablast cells in BLCA patients requires additional examination. We performed pathway enrichment analysis for 9 infiltrating immune cell types using three different methods, and the results showed certain similarities. Most of the pathways were downregulated in fibroblasts, endothelial cells, and epithelial cells, while a trend of upregulation was observed in the other 6 cell types, especially T cells, NK cells, and B cells. These findings suggest that infiltrating immune cells, particularly T cells, NK cells, and B cells, may play important roles in cellular functions and disease progression by positively regulating metabolic, signaling, and epigenetic pathways.

We identified FASN and RUNX2 as lactylation-associated hub genes that are significantly upregulated in BLCA and correlate with poor prognosis. Functional experiments demonstrated that silencing either gene in BLCA cell lines not only suppressed cell proliferation and induced apoptosis, but also led to a notable reduction in intracellular lactate levels. This decrease in lactate was accompanied by a corresponding decline in global protein lactylation, indicating that FASN and RUNX2 may act as upstream regulators of the lactate–lactylation axis.

Notably, Western blot analysis revealed that lactylation signals were most prominent at approximately 40 kDa, suggesting that lactylation in BLCA may primarily affect specific non-histone proteins within this molecular weight range. This finding shifts the focus away from a purely epigenetic framework toward a broader model in which lactylation may influence protein function, localization, or stability through direct post-translational modification. The biological significance of these ~40 kDa lactylated proteins warrants further investigation, particularly in relation to immune evasion, inflammatory signaling, or metabolic regulation in the tumor microenvironment.

Our single-cell RNA sequencing data further revealed that lactylation signatures were elevated in epithelial cells and tumor-infiltrating immune subsets, such as T cells and plasmablasts, suggesting that lactylation may contribute to reshaping the immune landscape in BLCA. Given that FASN and RUNX2 are also associated with immune-related pathways, including cytokine signaling and immune cell infiltration, their lactate-regulatory roles may exert multifaceted effects that go beyond intrinsic tumor growth.

Taken together, our findings propose a novel mechanistic axis—centered on FASN and RUNX2—linking metabolic enzymes to protein lactylation and downstream tumor-promoting processes. These insights not only expand our understanding of protein lactylation beyond histone modifications but also provide a rationale for targeting metabolic-epigenetic pathways in BLCA therapy. However, this study has limitations. Although we observed strong effects *in vitro*, *in vivo* validation and proteomic identification of lactylated substrates—especially those near 40 kDa—are essential to fully understand the biological roles of lactylation in BLCA. Future studies employing mass spectrometry and lactylation-specific enrichment strategies will be critical to uncover the functional targets and consequences of this modification in both tumor and immune contexts.

## Data Availability

The original contributions presented in the study are included in the article/**Supplementary Material**. Further inquiries can be directed to the corresponding author/s.

## Supplementary material

Supplementary Figure 1 Differential expression of 47 lactation-related differential genes in CD and control. **(A)** Volcano plot displaying gene expression differences; **(B)** Heatmap showing the expression of 47 differentially expressed genes in BLCA tissues compared to controls.

Supplementary Figure 2 Feature selection of lactylation-related genes. **(A)** LASSO regression narrowing 42 genes to 19 candidates; **(B)** Random Forest analysis of error rates versus tree numbers; **(C)** SVM-RFE showing cross-validation error by feature count, with 13 features optimal.

Supplementary Figure 3 Quality control metrics for RNA sequencing data. **(A)** Violin plots showing the distribution of quality control metrics, including RNA counts (nCount_RNA), feature counts (nFeature_RNA), mitochondrial content percentage (percent.mt), and hemoglobin content percentage (percent.HB) for the GSE222215 dataset before filtering; **(B)** Violin plots of the same quality control metrics after filtering, highlighting the improvement in data quality; **(C)** Scatter plot illustrating the correlation (r = 0.84) between RNA counts (nCount_RNA) and feature counts (nFeature_RNA) post-filtering.

Supplementary Figure 4 Heatmap of KEGG pathways. Each cell represents the pathway enrichment score based on the KEGG_MEDICUS subset of canonical pathways from the MSigDB database.

Supplementary Figure 5 Heatmap of PID pathways. Each cell represents the pathway enrichment score based on the PID subset of canonical pathways from the MSigDB database.

Supplementary Figure 6 Expression and knockdown validation of FASN and RUNX2 in BLCA cells. **(A)** Relative mRNA expression levels of AKR1B10, FASN, and RUNX2 in normal urothelial cells and various bladder cancer cell lines. B–C. qRT-PCR **(B)** and Western blot **(C)** validation of FASN knockdown in T24 and EJ-138 cells after siRNA transfection. **(D–E)** qRT-PCR **(D)** and Western blot **(E)** validation of RUNX2 knockdown in T24 and EJ-138 cells after siRNA transfection. All experiments were independently repeated at least three times. Data are presented as mean ± SD. *P < 0.05, **P < 0.01, ***P < 0.001, ****P < 0.0001, ns, not significant (one-way ANOVA with Tukey’s test).

Supplementary Figure 7 Quantitative analysis of proliferation and apoptosis following knockdown of FASN or RUNX2 in BLCA cells. **(A–B)** Quantification of EdU-positive cells in T24 and EJ-138 cells after transfection with siFASN **(A)** or siRUNX2 **(B)**. **(C–D)** Quantification of colony numbers following siFASN **(C)** or siRUNX2 **(D)** transfection. **(E–F)** Percentage of PI-positive cells in T24 and EJ-138 cells after knockdown of FASN **(E)** or RUNX2 **(F)**. **(G–H)** Apoptosis rates quantified by flow cytometry based on Annexin V-FITC/PI staining after siFASN **(G)** or siRUNX2 **(H)** treatment. All data are presented as mean ± SD from three independent experiments. *P < 0.05, **P < 0.01, ***P < 0.001, ****P < 0.0001, ns, not significant (one-way ANOVA with Tukey’s test).
