# Supplementary material for: Identification of lactylation-associated immune and metabolic regulators in bladder cancer via integrated bulk and single-cell transcriptomics
Source: Front Immunol. 2025 Jul 9;16:1604758. doi: 10.3389/fimmu.2025.1604758 (PMC12283629; doi:10.3389/fimmu.2025.1604758)
Supplement: Supplementary Figure 1 — Differential expression of 47 lactation-related differential genes in CD and control. (A) Volcano plot displaying gene expression differences; (B) Heatmap showing the expression of 47 differentially expressed genes in BLCA tissues compared to controls. [file DataSheet1.docx]

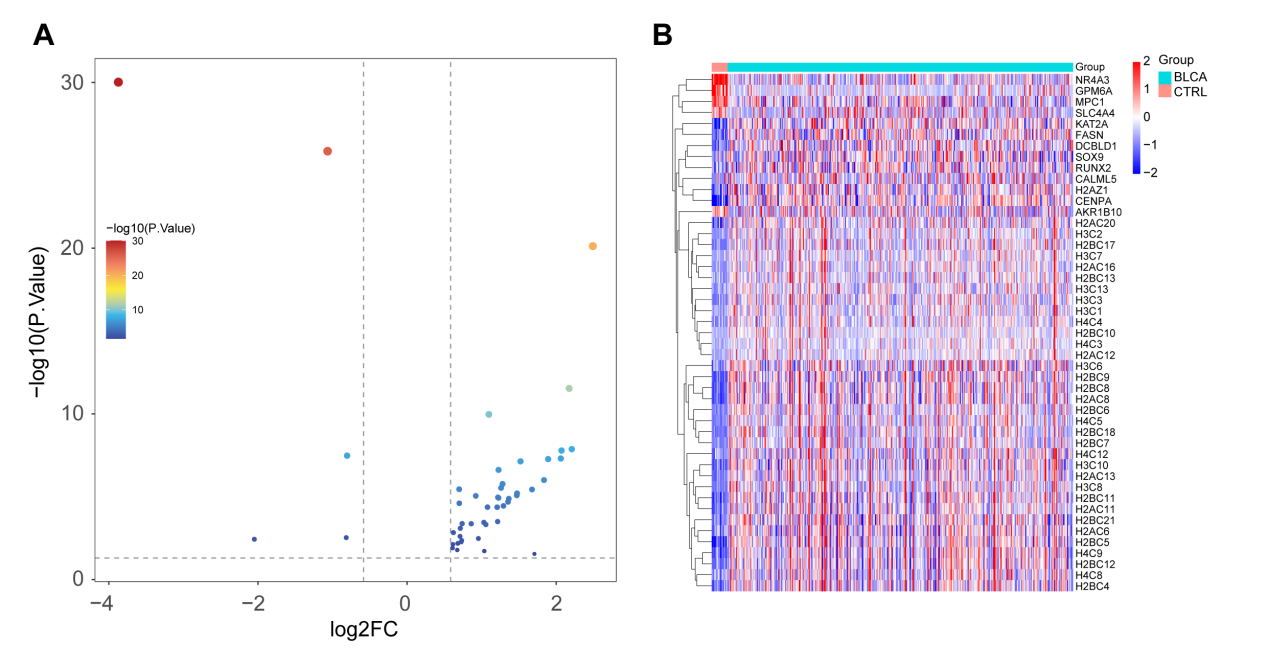


Supplementary Fig. 1 Differential expression of 47 lactation-related differential genes in CD and control.

A. Volcano plot displaying gene expression differences; B. Heatmap showing the expression of 47 differentially expressed genes in BLCA tissues compared to controls.


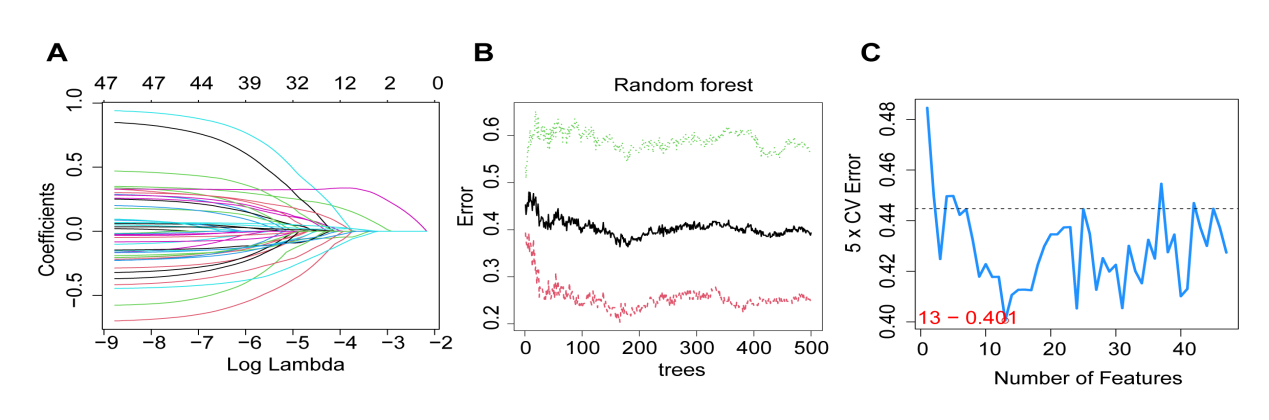


Supplementary Fig. 2 Feature selection of lactylation-related genes.

A. LASSO regression narrowing 42 genes to 19 candidates; B. Random Forest analysis of error rates versus tree numbers; C. SVM-RFE showing cross-validation error by feature count, with 13 features optimal.


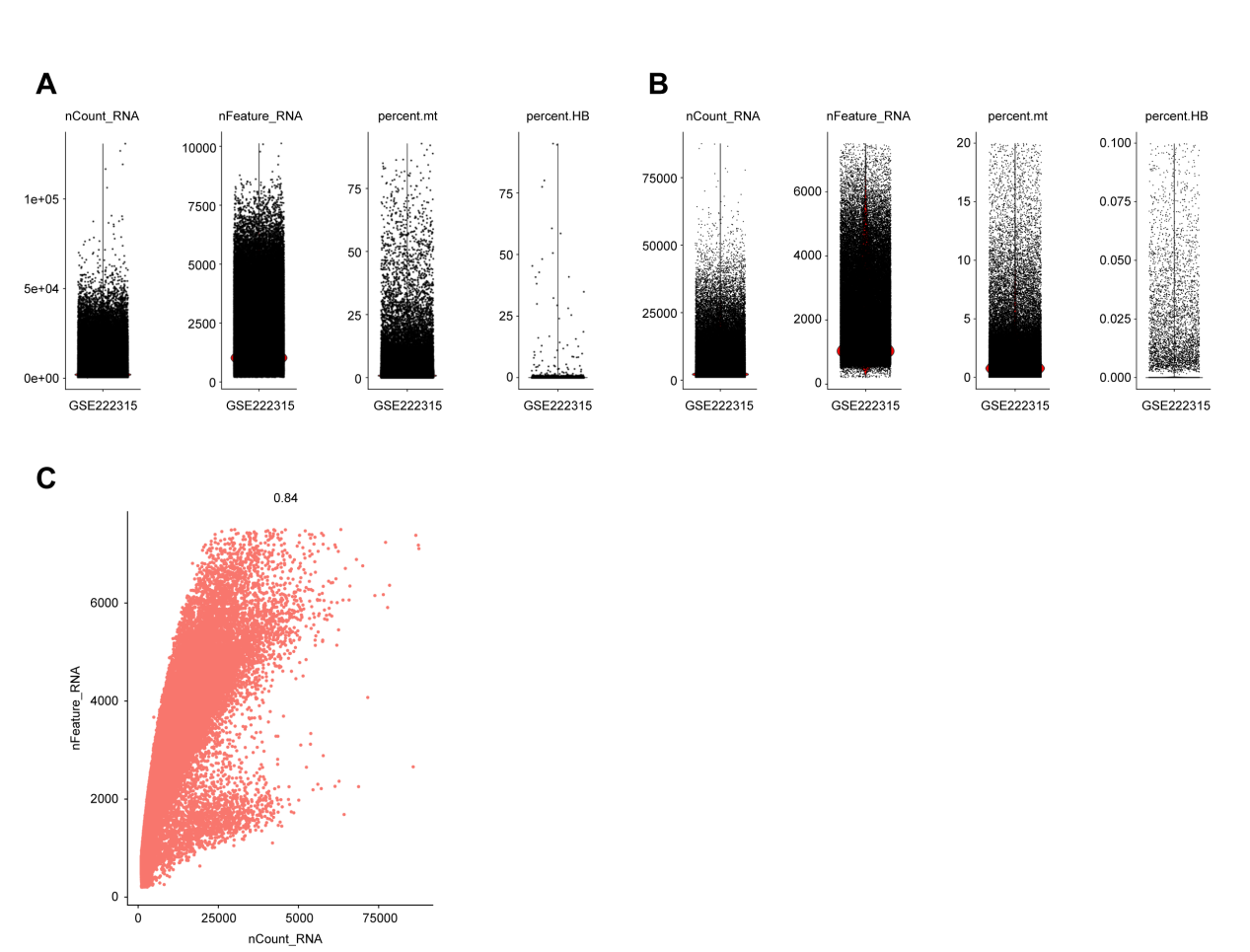


Supplementary Fig. 3 Quality control metrics for RNA sequencing data.

A. Violin plots showing the distribution of quality control metrics, including RNA counts (nCount_RNA), feature counts (nFeature_RNA), mitochondrial content percentage (percent.mt), and hemoglobin content percentage (percent.HB) for the GSE222215 dataset before filtering; B. Violin plots of the same quality control metrics after filtering, highlighting the improvement in data quality; C. Scatter plot illustrating the correlation (r = 0.84) between RNA counts (nCount_RNA) and feature counts (nFeature_RNA) post-filtering.


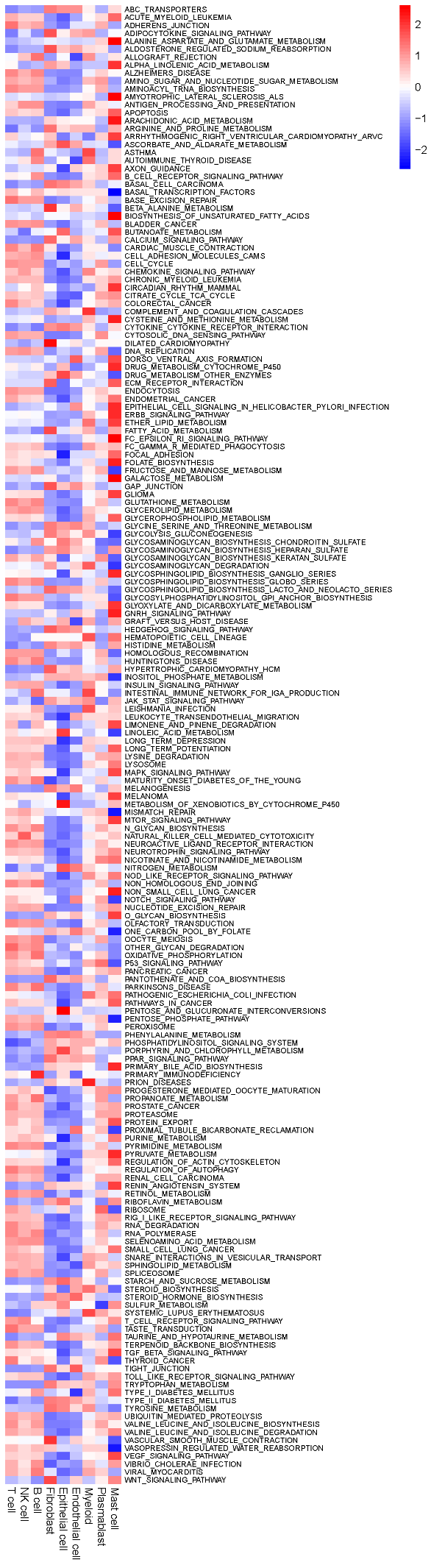


Supplementary Fig. 4 Heatmap of KEGG pathways.

Each cell represents the pathway enrichment score based on the KEGG_MEDICUS subset of canonical pathways from the MSigDB database.


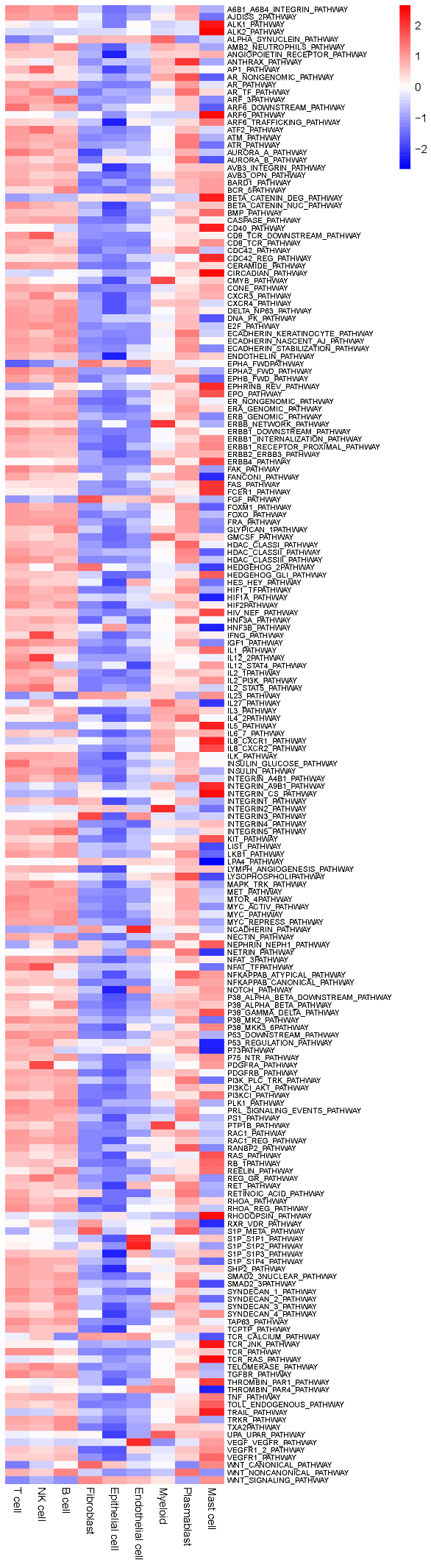


Supplementary Fig. 5 Heatmap of PID pathways

Each cell represents the pathway enrichment score based on the PID subset of canonical pathways from the MSigDB database.


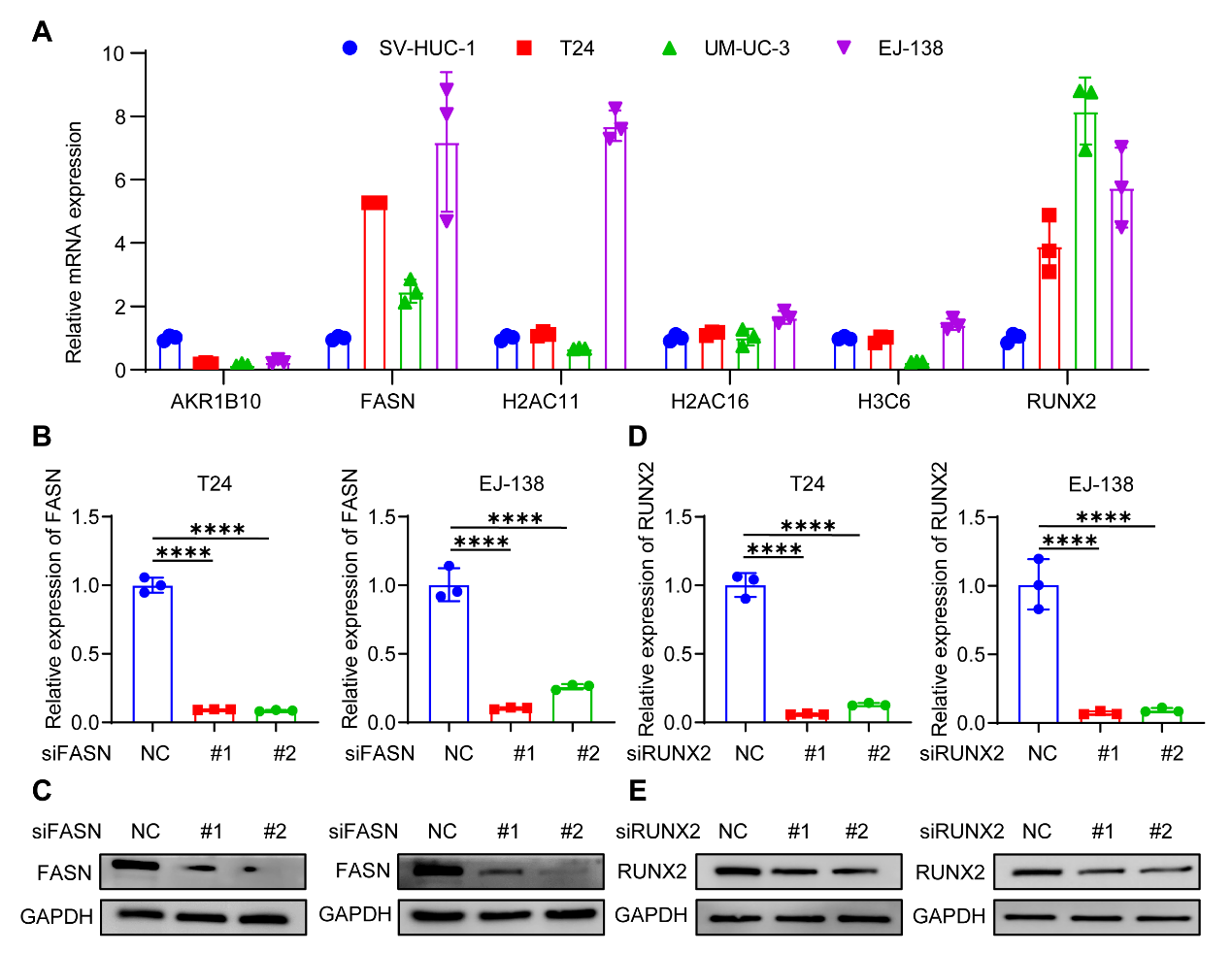


Supplementary Fig. 6 Expression and knockdown validation of FASN and RUNX2 in BLCA cells.

A. Relative mRNA expression levels of AKR1B10, FASN, and RUNX2 in normal urothelial cells and various bladder cancer cell lines. B–C. qRT-PCR (B) and Western blot (C) validation of FASN knockdown in T24 and EJ-138 cells after siRNA transfection. D–E. qRT-PCR (D) and Western blot (E) validation of RUNX2 knockdown in T24 and EJ-138 cells after siRNA transfection. All experiments were independently repeated at least three times. Data are presented as mean ± SD. *P < 0.05, **P < 0.01, ***P < 0.001, ****P < 0.0001, ns, not significant (one-way ANOVA with Tukey’s test).
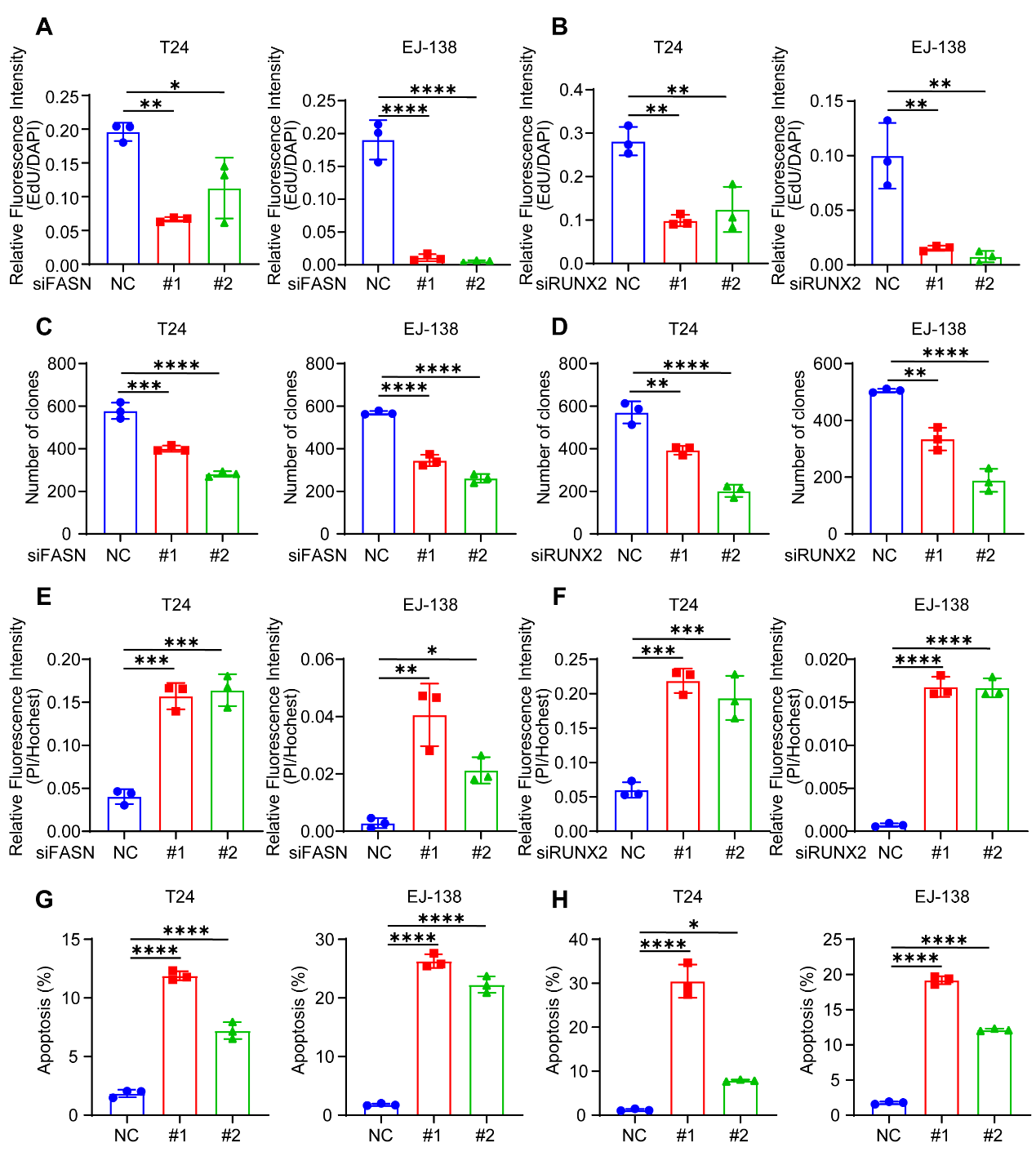


Supplementary Figure 7. Quantitative analysis of proliferation and apoptosis following knockdown of FASN or RUNX2 in BLCA cells.

A–B. Quantification of EdU-positive cells in T24 and EJ-138 cells after transfection with siFASN (A) or siRUNX2 (B). C–D. Quantification of colony numbers following siFASN (C) or siRUNX2 (D) transfection. E–F. Percentage of PI-positive cells in T24 and EJ-138 cells after knockdown of FASN (E) or RUNX2 (F). G–H. Apoptosis rates quantified by flow cytometry based on Annexin V-FITC/PI staining after siFASN (G) or siRUNX2 (H) treatment. All data are presented as mean ± SD from three independent experiments. *P < 0.05, **P < 0.01, ***P < 0.001, ****P < 0.0001, ns, not significant (one-way ANOVA with Tukey’s test).
